# George K. Amerman, M. D.

**Published:** 1867-09

**Authors:** 


### *George K. Amerman, M.D.*

At a meeting of the Chicago Medical Society, held at the Court House, June 28, Dr. J. P. Ross announced to the Society that a brother member, Dr. GEORGE K. AMERMAN, had departed this life, at Marcellus, N. Y., June 20, 1867. For nearly two years his health had been failing, and in April he had abandoned the practice of his profession, going home to die of consumption. Dr. Ross moved the appointment of a committee to prepare a series of resolutions appropriate to this sorrowful occasion. The motion having prevailed, the following gentlemen were appointed by the Chairman: Drs. Ross,

Holmes, Bevan, Heydock, and Marguerat. They reported as follows:

Having been informed of the death of their associate, George K. Amerman, M.D., the members of the Chicago Medical Society desire to testify for the deceased their respect, and their feelings of personal loss by these resolutions:

*First.* That in this affliction we lament the death of one who, long identified with our community, though young in years, was old in professional renown. A man whose life was a career of brilliant success, a Christian in deed, as well as in name, at the height of his reputation, he has now received the crown of immortality.

*Second.* That to the members of our profession we earnestly commend the example afforded by the life of our departed associate.

*Third.* That to the surviving relatives of our beloved friend we tender this expression of our sympathy in view of their bereavement, ever desiring with them to bow in humble acknowledgment of the almighty power of that God in whose hand are the issues of life and of death.

*Fourth.* That a copy of these resolutions be furnished to the relatives of the deceased, and to the medical journals and daily newspapers of the city.

The report of the Committee was accepted.

Dr. Ross then read a technical history of the last illness of the departed brother.

Dr. J. R. Gore, who had known Dr. AMERMAN from infancy, being invited to address the Society, proceeded to relate the following interesting facts concerning his friend:

At the time of his death Dr. Amerman was in his thirty-fifth year. The son of a rural farmer in Central New York, his boyhood was distinguished by fondness for study, and by disinclination to the drudgery of farm work. He was obedient and kind, but *good for nothing* on the farm. He early manifested a passion for teaching, and at the age of sixteen his only ambition was to become a school-teacher. His father, however, proposed that he should study medicine. To this he consented. Placed under the guidance of Dr. Gore, then a practicing physician in Owasco, N. Y., he pledged himself to pursue the study of medicine, subject to the direction of his preceptor, for five years before attempting to commence its practice. He accordingly attended three courses of lectures in the University Medical College of New York, graduating with honor in the spring of 1854. Still acting under the guidance of his preceptor, he secured a position for one year on the resident medical staff of Bellevue Hospital. The next year he became a member of the resident surgical staff of the same hospital. There, in the enjoyment of the friendship and confidence of the most distinguished physicians and surgeons of New York City, Dr. Amerman completed the fifth year of his course of study. At once received into partnership by his former preceptor, and removing to Chicago, he commenced the practice of his profession in 1856. He was appointed Surgeon to the Illinois Central Railroad, an office which he filled with great credit until the time of his death. He was also one of the Surgeons to the City Hospital, until that institution was closed during the war, and was the leading spirit in the surgical staff of the County Hospital from the time of its renovation until failing health compelled him to surrender his large and lucrative practice.

After listening to the remarks of different gentlemen who united in eulogizing the memory of the deceased, the resolutions were adopted, and the Society adjourned.

J. P. ROSS, M.D., President.

HENRY M. LYMAN, M.D., Secretary.

ACTION OF THE HOSPITAL BOARD.

At a meeting of the Medical Board of the County Hospital, Chicago, to take action on the death of Dr. GEORGE K. AMERMAN, the following resolutions were adopted:

*Resolved,* That the members of this Board have heard, with feelings of the deepest sorrow, the death of their colleague, Dr. Amerman, and desire to put on record their high appreciation of him as a gentleman and a surgeon.

*Resolved,* That in his death the staff of this Hospital has lost a most efficient member, and one most intimately identified with its best interests; that the profession at large has lost one of its brightest ornaments, and our community a most valuable citizen.

*Resolved,* That we believe his death was the result of too arduous devotion to his chosen calling, and that he has added another to that long list of glorious martyrs who have given up their lives in the cause of suffering humanity.

*Resolved,* That a copy of these resolutions be sent to his friends at the East, and to the public journals.

| | |
|---|---|
| R. C. HAMILL, M.D., | H. A. JOHNSON, M.D., |
| J. P. ROSS, M.D., | R. G. BOGUE, M.D., |
| H. W. JONES, M.D., | CHARLES G. SMITH, M.D., |
| THOS. BEVAN, M.D., | H. M. LYMAN, M.D., |
| J. R. GORE, M.D., | J. S. HILDRETH, M.D. |

